# Simple and Rapid Quality Control of Sulfated Glycans by a Fluorescence Sensor Assay—Exemplarily Developed for the Sulfated Polysaccharides from Red Algae *Delesseria sanguinea*

**DOI:** 10.3390/md12042205

**Published:** 2014-04-10

**Authors:** Susanne Lühn, Juliane C. Grimm, Susanne Alban

**Affiliations:** Pharmaceutical Institute, Pharmaceutical Biology, Christian-Albrechts-University of Kiel, Gutenbergstr. 76, D-24118 Kiel, Germany; E-Mails: sluehn@pharmazie.uni-kiel.de (S.L.); jgrimm@pharmazie.uni-kiel.de (J.C.G.)

**Keywords:** *Delesseria sanguinea*, sulfated polysaccharides, quality control, fluorescence sensor, (red) algae, biopolymer, assay development, Polymer-H, sulfated glycan, fondaparinux

## Abstract

Sulfated polysaccharides (SP) from algae are of great interest due to their manifold biological activities. Obstacles to commercial (especially medical) application include considerable variability and complex chemical composition making the analysis and the quality control challenging. The aim of this study was to evaluate a simple microplate assay for screening the quality of SP. It is based on the fluorescence intensity (FI) increase of the sensor molecule Polymer-H by SP and was originally developed for direct quantification of SP. Exemplarily, 65 SP batches isolated from the red alga *Delesseria sanguinea* (*D.s.*-SP) and several other algae polysaccharides were investigated. Their FI increase in the Polymer-H assay was compared with other analytical parameters. By testing just one concentration of a *D.s.*-SP sample, quality deviations from the reference *D.s.*-SP and thus both batch-to-batch variability and stability can be detected. Further, structurally distinct SP showed to differ in their concentration-dependent FI profiles. By using corresponding reference compounds, the Polymer-H assay is therefore applicable as identification assay with high negative predictability. In conclusion, the Polymer-H assay showed to represent not only a simple method for quantification, but also for characterization identification and differentiation of SP of marine origin.

## 1. Introduction

Sulfated polysaccharides (SP) represent an important class of biopolymers. In vertebrates, sulfated glycosaminoglycans occur as components of the extracellular matrix and the glycocalyx and exhibit multiple (patho-)physiological functions [1,2,3,4]. Heparin, one of these glycosaminoglycans, is used for more than seven decades as antithrombotic drug. Intense heparin research and the increasing functional understanding of glycosaminoglycan-protein interactions have stimulated the development of heparin/heparin sulfate-based or glycosaminoglycan mimetics for use as anti-angiogenic, anti-metastatic, anti-inflammatory, anticoagulant, and anti-thrombotic agents [5].

Another even larger source of SP are marine algae and marine invertebrates. Marine SP include galactans, fucoidans, glycosaminoglycans, glucans, and some heteropolysaccharides and feature a tremendous biodiversity [6,7]. The knowledge about their physiological functions is limited with few exceptions like the role of fucans in sea urchin fertilization and the participation of SP in the structural assemblies of the body walls of ascidians and sea cucumbers [8,9]. The virtually ubiquitous occurrence in the cell walls and intercellular spaces of macroalgae is supposed to be an adaption to the high salinity and mechanical stress of their habitat [10]. Carrageenans and agarans, two types of sulfated galactans extracted from red algae species, are important hydrocolloids widely used as texturing agents and stabilizers in food, cosmetics, pharmaceuticals and for some other applications [11,12]. Besides such industrial-scale applications, marine SP, such as fucoidans, exhibit a wide range of biological activities so that they are considered to be promising candidates for numerous therapeutic applications [13,14]. However, despite intense research during the last two decades, there is so far no approved medicinal product with marine SP as drug substances and they are currently only utilized as ingredients in food supplements and cosmetics [15,16]. Critical issues for SP are the high requirements on the pharmaceutical quality of medicinal products. Marine SP are complex, heterogeneous molecule mixtures, which additionally substantially vary in their composition depending on the source material (e.g., alga species, time of harvest), environmental parameters (e.g., light, nutrition, salinity, temperature), as well as the process of extraction and purification, which may impact their pharmacological activities and consequently also their efficacy and safety [14,17,18,19]. Since besides efficacy and safety guaranteed pharmaceutical quality is a *conditio sine qua non* for drug approval, marine SP with defined and reproducible composition have to be developed to increase the chances of medical applications.

For the SP from a specific alga, the respective influencing parameters have to be identified and the production procedure has to be individually optimized and standardized. By isolating more than 250 SP batches from about 30 alga batches, meanwhile we performed such work on the SP fraction from the red alga *Delesseria sanguinea* (*D.s.*-SP) having an interesting pharmacological profile [17,18,20].

Although the “process defines the product”, a standardized procedure alone is not sufficient and suitable methods are needed to control and ensure the quality of a SP. In contrast to chemically defined small molecules, the quality control of SP of biological origin is, challenging due to their structural characteristics as well as due to a certain unavoidable batch-to-batch variability. This was illustrated by the heparin scandal in 2008, where counterfeit heparin caused severe adverse events including cases of death. Heparin contaminated with oversulfated chondroitin sulfate, a cheap semi-synthetic heparin substitute, even passed the quality control according to the actual heparin monographs of the Pharmacopoeias and was only detected by complex pharmaceutical analysis [21].

To elucidate the structural composition of SP, a combination of high-sophisticated methods like gas-liquid chromatography-mass spectrometry (GC-MS) and various nuclear magnetic resonance spectroscopy (NMR) techniques is required [22]. However, such methods are not suitable for routine quality control of SP. The testing of SP used as pharmaceutical excipients (e.g., agar, carrageenans) is, therefore, limited to nonspecific characteristics important for their respective application, such as solubility, viscosity, and gel formation. Moreover, it can be assumed that there are many products containing marine SP, whose identity and structural composition is not routinely controlled at all. Consequently, there is a demand for feasible and non-expensive methods for an adequate quality control of SP to ensure the claimed effects and the safety of such products.

In the analytics, fluorescent sensors are considered to be promising tools [23]. Even so, for SP several fluorescent sensor molecules have been described [24].

Recently, we developed a sensitive microplate assay for quantification of heparins, which is based on the fluorescence intensity (FI) increase of the heparin sensor Polymer-H [25]. Polymer-H consists of a methacrylamide skeleton linked with three functional units: (1) *ortho*-aminomethylphenylboronic acid units forming boronic esters with glycans; (2) ethylammonium moieties electrostatically binding to anionic sulfated glycans; and (3) fluorescent dansyl monomers [26]. In the so-called Polymer-H assay, FI of the sensor proved to be concentration-dependently amplified in the presence of heparins, other glycosaminoglycans and semi-synthetic sulfated glucans. The extent of the FI increase showed to be mainly dependent on the degree of sulfation, whereas molecular weight and glycan structure of the examined series of heparins and semi-synthetic glucan sulfates played only minor parts.

The first aim of the presented study was to work out whether the fluorescent sensor Polymer-H can also be used to detect and quantify sulfated algae polysaccharides like the *D.s.*-SP, which considerably differ in their degree of sulfation, molecular mass and glycan structure from the SP tested so far. Next, regarding the importance of a reproducible quality of SP, which usually requires comprehensive analytics, the suitability of the Polymer-H assay should be evaluated as a simple and rapid screening method for the quality control of SP. For this, a total of 65 different *D.s.*-SP batches were tested in the Polymer-H assay. The samples included batches isolated from *Delesseria sanguinea* harvested at different locations, at different seasons and years, as well as samples obtained by various extraction procedures. The Polymer-H assay results were compared with other analytical characteristics of the batches to assess the impact of various quality parameters on the Polymer-H assay. By means of the obtained data, FI increase limits for an adequate *D.s.*-SP quality were established. Finally, it should be examined, whether the Polymer-H assay can be utilized as identification assay for *D.s.*-SP and to distinguish them from other sulfated glycans.

## 2. Results and Discussion

### 2.1. Polymer-H Assay for Detection and Quantification of D.s.-SP

The Polymer-H assay was originally developed for quantification of heparins with relatively high sulfate contents (about 30%) and degrees of sulfation (about 1.2), respectively. It proved to detect a semi-synthetic linear homoglucan sulfate with a degree of sulfation as low as 0.60, but not another one with a still lower degree of sulfation of 0.25 [25]. The FI increase of Polymer-H by the sulfated homoglucan with degree of sulfation of 0.60 was about four times lower than that by heparin and a homoglucan sulfate with a degree of sulfation of 1.26. As the *D.s.*-SP have considerably lower sulfate contents (average 20.4% ± 1.6%), it should be initially clarified, whether *D.s.*-SP as an example of SP from algae can be quantified with Polymer-H at all. The, thus far, investigated compounds were mostly heparins and semi-synthetic linear glucan sulfates, whereas *D.s.*-SP consist of a homogenous fraction of branched sulfated xylogalactans [17,18]. Therefore, the question arose whether this branching has any influence on the tendency of SP to bind to Polymer-H.

#### 2.1.1. Applicability of the Polymer-H Assay

**Figure 1:**
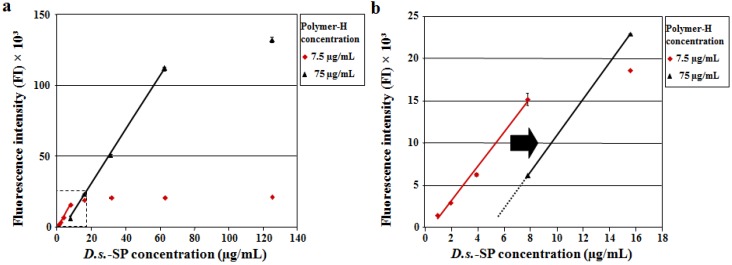
(**a**,**b**) Concentration-dependent fluorescence intensity (FI) increasing effect of a typical *D.s.*-SP (dissolved in double distilled water) on Polymer-H. The red line results from 7.5 µg/mL Polymer-H in the assay solution (*y* = 1923*x* − 8597, *r* = 0.9997), the black one from 75 µg/mL (*y* = 2032*x* − 985, *r* = 0.9966). At the tenfold Polymer-H concentration, the linear range is parallel shifted to higher *D.s.*-SP concentrations.

**Figure 2:**
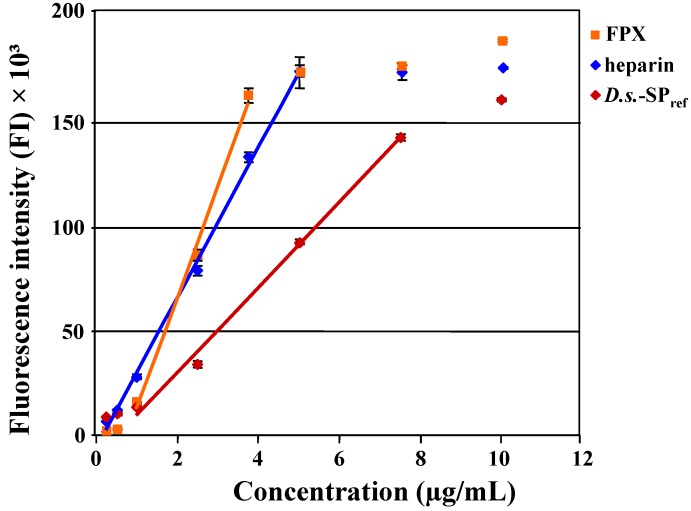
Fluorescence intensity (FI) increase of Polymer-H (7.5 µg/mL in 0.9% NaCl) by fondaparinux (FPX; *y* = 53524*x* − 40835, *r* = 0.997), heparin (*y* = 36244*x* − 6402, *r* = 0.999) and *D.s.*-SP_ref_ (*y* = 20500*x* − 11003, *r* = 0.997).

First, a typical *D.s*.-SP (1–125 µg/mL) with a degree of sulfation of 0.77 and an elastase inhibitory activity (IC_50_) of 0.336 ± 0.003 µg/mL was examined in the Polymer-H assay performed as developed for the heparin quantification using a Polymer-H concentration of 7.5 µg/mL. From 1.0 to 7.8 µg/mL *D.s.*-SP caused a linear FI increase of Polymer-H followed by a plateau up to 125 µg/mL (Figure 1). Thus, the branched sulfated xylogalactan chains of *D.s.*-SP seem to interact with the aminomethylphenylboronic acid units of Polymer-H similar to the sulfated glycosaminoglycan chains of heparin, maybe by forming boronic esters [25,27]. The linear range only slightly differed from that of heparin (0.3 to 5.0 µg/mL) (Figure 2 and [25]). According to the known degree of sulfation dependence the slope of the line was smaller and the maximum FI increase was lower due to the lower degrees of sulfation of *D.s*.-SP indicating weaker electrostatic interactions with the ethylammonium moieties of Polymer-H [25,26]. Interestingly, the effect of *D.s.*-SP on Polymer-H was stronger than assumed from the glucan sulfates with degree of sulfation of 0.6 and 1.26 mentioned above (Figure 2 and [25]) suggesting that the branched glycan structure of *D.s.*-SP does not impair, but rather improves the binding to Polymer-H, maybe due to additional interactions of the xylose side chains of *D.s.*-SP with the aminomethylphenylboronic acid units of the Polymer-H. Consequently, the extent of the FI increasing effect on Polymer-H seems to be an individual parameter of a SP being not exclusively dependent on its degree of sulfation.

A tenfold higher Polymer-H concentration (75 µg/mL) led to a parallel shift of the respective linear range of the *D.s.*-SP to the tenfold concentration (7.8 to 62.5 µg/mL) and a corresponding stronger FI increase, whereas the accuracy was similar to that at 7.5 µg/mL (Figure 1a,b). This implies that the linear range, which comprises about one order of magnitude, can be aligned to a given SP concentration by variation of the Polymer-H concentration.

To sum up, the Polymer-H assay showed to be suitable for the detection and quantification of algae-derived SP like *D.s.*-SP, but as the interactions between any SP and Polymer-H are obviously not only dependent on its degree of sulfation, initially the individual intrinsic FI increasing effect of the respective SP has to be evaluated.

The following experiments were performed with a final Polymer-H concentration of 7.5 µg/mL and SP concentrations ranging from 1.0 to 10 µg/mL.

#### 2.1.2. Selection of Calibrator and Reference Substance

To apply the Polymer-H assay for quantification, batch control (see Section 2.2) and identification (see Section 2.3) of *D.s.*-SP or other SP, suitable calibrators and reference substances are needed for concurrent testing on every microplate.

As reference substance a high quality *D.s.*-SP batch or accordingly a representative batch of the respective SP should be selected. The chosen batch *D.s.*-SP_ref_ was extracted according to the final extraction procedure. The corresponding *D.s.* batch was from *D.s.* harvested in April 2008 at the “Artificial Reef Nienhagen” (in spring lowest content of floridean starch) and manually purified before extraction. It contains 20.5% sulfate, 4.9% floridean starch, and 10.5% protein and has a relatively strong elastase inhibitory activity (IC_50_ = 0.186 ± 0.004 µg/mL).

*D.s.*-SP_ref_ serves as reference substance in all *D.s.*-SP related applications of the Polymer-H assay and is additionally used as calibrator for quantification. Alternatively, depending on the purpose of the quantification, the respective *D.s.*-SP batch can be used for calibration.

Furthermore, we intended to introduce a general calibrator making it possible to compare the results of assay runs independent of the reference compound for the respective SP of interest. Such a compound should be a sulfated glycan causing a reproducible, batch-independent FI increase of Polymer-H and unlimited available. An attractive candidate is fondaparinux (FPX), a synthetic pentasaccharide containing 8 sulfate groups (*i.e.*, degree of sulfation 1.6) corresponding to the antithrombin binding site of heparin, which is approved as antithrombotic drug since 2001 [28]. In contrast to sulfated glycans of natural origin, fondaparinux is chemically defined and therefore available in constant quality. In the Polymer-H assay, this high sulfated glycan proved to lead to high and reproducible FI increases [29]. A further advantage of the calibrator fondaparinux is that it can also be used for investigations of other SP in the Polymer-H assay.

Figure 2 shows the concentration-dependent FI increases of Polymer-H in the presence of fondaparinux, heparin and *D.s.*-SP_ref_. The extent of the effects degree of sulfation-dependently increased in the order *D.s.*-SP_ref_ < heparin < fondaparinux, whereas the concentration range of linear FI increase inversely increased, *i.e.*, *D.s.*-SP_ref_ (1.0 to 7.5 µg/mL) > heparin (0.3 to 5.0 µg/mL) > fondaparinux (1.0 to 3.8 µg/mL).

The use of fondaparinux as calibrator means that the FI increase by 3.75 µg/mL fondaparinux, the highest concentration within the linear range, was defined as 100% FI increase and the FI of Polymer-H itself represented 0 FI increase. Related to these values, the FI increase by 7.5 µg/mL *D.s.*-SP_ref_ amounted to 92.2% (Figure 2).

The intra- and inter-assay variability demonstrate the suitability of this approach: The mean intra-assay coefficients of variation of the FI increase values were 3.27% for 3.75 µg/mL fondaparinux (max. 4.24%) and 3.23% for 7.5 µg/mL *D.s.*-SP_ref_ (max. 4.95%); the inter-assay coefficients of variation (assays at 7 different days) were 4.28% for fondaparinux and 7.48% for *D.s.*-SP_ref_. When the results of *D.s.*-SP_ref_ were transformed into % values related to fondaparinux, the mean intra-assay coefficient of variation was 3.32% (max. 4.95%) and the inter-assay coefficient of variation was 7.98%.

In the experiments hereinafter presented, *D.s.*-SP_ref_ was used as reference substance (1.0 to 10.0 µg/mL or at least 7.5 µg/mL) and fondaparinux as calibrator (0.25 to 3.75 µg/mL or at least 3.75 µg/mL). For comparison of various *D.s.*-SP batches, the FI increases by 7.5 µg/mL *D.s.*-SP were transformed into %-values related to the FI increase by 3.75 µg/mL fondaparinux (100%).

### 2.2. Polymer-H Assay for the Quality Control of D.s.-SP Evaluated by Means of a Large Series of D.s.-SP Batches

#### 2.2.1. Investigation of *D.s.*-SP Isolated from *D.s.* Harvested at Different Habitats

The location of an alga with its specific environmental parameters is known to contribute to the diversity of its chemical composition [19]. To investigate whether the habitat of *D.s.* has an influence on the composition of the extractable SP, *D.s.*-SP was isolated from algae material collected at different habitats in April 2008: Reef Nienhagen, Baltic Sea (*D.s.*-SP 1), Bay of Kiel, Baltic Sea (*D.s.*-SP 2), Helgoland, North Sea (*D.s.*-SP 3) and Roscoff, North Atlantic (*D.s.*-SP 4). As the *D.s.* batches were distinctly populated by various epibionts, they were extracted both as harvested (indicated as *D.s.*-SP “−”) and after manual purification (indicated as *D.s.*-SP “+”). The eight obtained *D.s.*-SP batches turned out to vary in some analytical and pharmacological characteristics.

**Figure 3:**
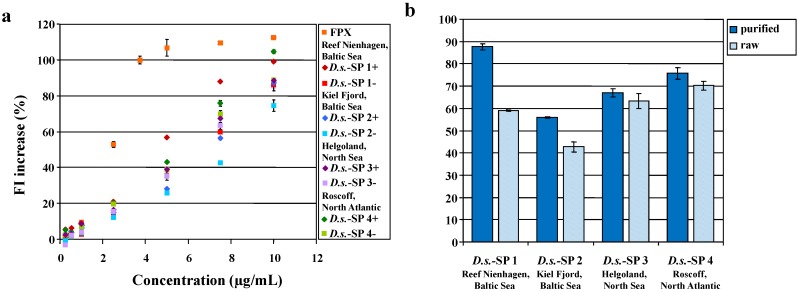
(**a**,**b**) Fluorescence intensity (FI) increase of Polymer-H (7.5 µg/mL in 0.9% NaCl) by *D.s.*-SP batches isolated from *Delesseria sanguinea* harvested at different habitats. The FI increase is indicated as percentage related to the FI increase by 3.75 µg/mL fondaparinux (FPX, 100%). *D.s.*-SP batches signed with “−” means that the algae were extracted as harvested, whereas “+” means that the algae were manually purified from epibionts before extraction. (**a**) Concentration-dependent effects of the eight *D.s.*-SP batches; (**b**) comparison of the “+” and “−” *D.s.*-SP batches by their effects at 7.5 µg/mL.

Therefore, these batches were thought to be a good example to evaluate the Polymer-H assay as a screening method for the quality control of sulfated glycans. As shown in Figure 3a, the *D.s.*-SP batches indeed differed in their concentration-dependent FI increasing effects on Polymer-H, which was most pronounced at 7.5 µg/mL *D.s.*-SP. The FI increases by the *D.s.*-SP “+” from purified batches were higher than those by the corresponding “−” *D.s.*-SP batches, whereby the extent differed in dependence on the amount and type (algae, bryozoan, mussels) of epibionts. Overall, the FI increases by the *D.s.*-SP batches seemed to show a positive correlation with their respective sulfate contents (*r* = 0.49), whereas there seemed to be a rough trend of an inverse correlation between FI increases and protein contents (*r* = 0.20).

#### 2.2.2. Influence of Sulfate and Protein Content of 57 *D.s.*-SP Batches on Their FI Increasing Effect

Based on the findings with the *D.s.*-SP batches isolated from *D.s.* growing at different habitats, the suggested impact of sulfate and protein content should be examined by measuring a larger series of *D.s.*-SP batches (7.5 µg/mL) in the Polymer-H assay. For this, 57 *D.s.*-SP batches were selected, which were intentionally heterogeneous. The sample pool included not only batches isolated from *D.s.* harvested at different seasons and years, but also batches not obtained by the finally optimized extraction procedure. Their sulfate contents ranged from 3.9% to 22.4% and their protein contents from 4.8% to 35.6%.

**Figure 4:**
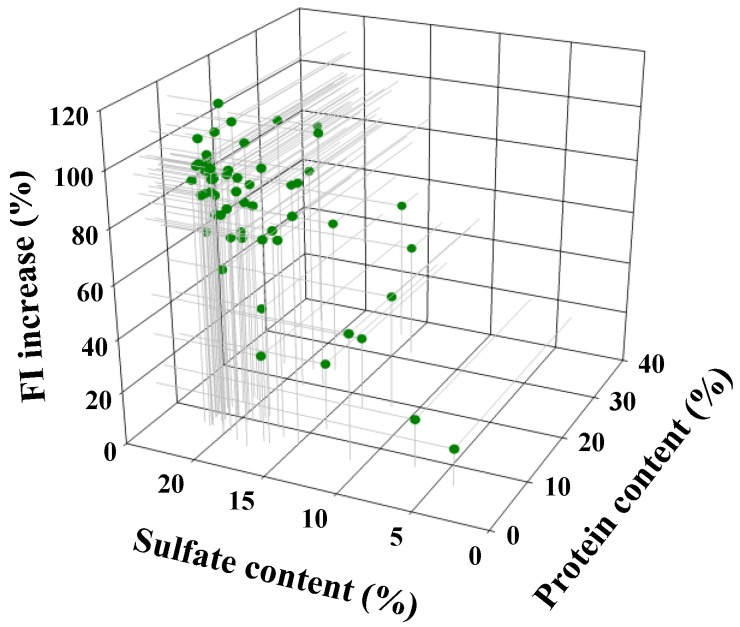
Fluorescence intensity (FI) increase of Polymer-H (7.5 µg/mL in 0.9% NaCl) in dependence of the sulfate and the protein content of 57 heterogenic *D.s.*-SP batches. The FI increase induced by 7.5 µg/mL of the *D.s.*-SP batches is indicated as percentage related to the FI increase by 3.75 µg/mL fondaparinux (100%).

Data analysis revealed a good correlation between the FI increase and the sulfate content of the *D.s.*-SP (*r* = 0.78), whereas there was no dependence of the FI increases on their protein content (*r* = 0.16). The FI values increased with increasing sulfate content irrespectively of the protein content of the batches (Figure 4).

The dependence of the FI increase on the sulfate content of the *D.s.*-SP is in line with its known degree of sulfation dependence [25]. However, it has to be considered that the sulfate content of the *D.s.*-SP batches rather represents a quality parameter than a structural characteristic of the *D.s.*-SP. The degree of sulfation of the pure xylogalactans showed to be consistently 0.73 ± 0.08. In contrast, the sulfate content of the *D.s.*-SP relates to the entire batch, which may contain more or less protein, floridean starch and possibly any other components depending on the quality of the extracted algae material and the applied isolation procedure. Accordingly, the sulfate content of a *D.s.*-SP batch corresponds to its xylogalactan content. The found correlation between the quality parameter sulfate content and the FI increase is therefore judged as an argument for the suitability of the Polymer-H assay for the quality control of *D.s.*-SP.

In addition, the FI measurements of the *D.s.*-SP pool turned out to correlate with their elastase inhibitory activity (IC_50_ ranging from 0.113 to 1.201 µg/mL, *r* = 0.80), which represents a proven surrogate parameter for the quality of *D.s.*-SP [17,18,20].

#### 2.2.3. Polymer-H Assay to Check *D.s.*-SP for Instability, Contamination and Co-Extracted Starch

Sulfated glycans like the *D.s.*-SP are known to be instable under acid conditions due to desulfation and subsequent degradation. For example, this can occur when a SP is excessively dialyzed and then lyophilized without neutralization of the solution. By means of the sulfate content, such processes can only be recognized if the sample is dialyzed and lyophilized before the elementary analysis. Therefore, it should be examined whether the Polymer-H assay detects such instability. For this, two *D.s.*-SP batches with sulfate contents of 21.4% and 17.4%, respectively, and calculated degree of sulfation values of 0.83 and 0.77 were partially desulfated. Compared with the intact *D.s.*-SP, the FI increases of Polymer-H were 70% and 87%, respectively, lower. Elementary analysis revealed decreased sulfate contents of 14.3% and 13.5% (degrees of sulfation of 0.47 and 0.51), respectively, proving the desulfation. In this experiment, the FI response correlated with the degrees of sulfation of the xylogalactans. Since unbound sulfate ions showed to have no influence on the FI of Polymer-H [25]. Consequently, in contrast to the elementary analysis, the Polymer-H assay proved to be additionally suitable to control the stability of *D.s.*-SP and other SP, whereas this is not possible by elementary analysis unless prior excessive dialyzes and lyophilisation of the SP.

As shown in Figure 3, manual purification of *Delesseria sanguinea* from epibionts before extraction can result in considerable increase of the FI in the Polymer-H assay. This was confirmed with two other *D.s.*-SP pairs isolated from purified and non-purified *D.s.*, respectively (80.7% ± 0.2% *vs.* 53.9% ± 0.2% and 89.6% ± 0.6% *vs.* 79.4% ± 0.4%). The reason for these findings is that *Delesseria sanguinea* growing at the “Artifical Reef Nienhagen” is nearly free of epiphytes, but depending on the time of harvest it can be strongly populated by *Mytilus* *edulis*. Extraction revealed that this type of mussels does not contain extractable SP, but results in increased protein content of the extracted *D.s.*-SP and, thus, in “dilution” of the sulfated xylogalactans. Consequently, the Polymer-H assay can be used to check whether the *D.s.*-SP was isolated from algae material contaminated with *Mytilus* *edulis.*

As learned from investigations to optimize the extraction procedure, the *D.s.*-SP yields obtained by multiple short-term extractions of the algae material each performed with fresh extraction solvent are higher than those of one long-term extraction. Usually, the 1st and 2nd extracts do not significantly differ in their composition, but the subsequent ones may contain increased contents of glucose (*i.e.*, floridean starch) depending on the used algae material. To avoid a loss of quality, these parameters have to be checked before combining the partial batches of multiple extractions.

Examination of *D.s.*-SP batches obtained by multiple extractions in the Polymer-H assay revealed that the FI increase is the lower, the higher the glucose (*i.e.*, floridean starch) content of *D.s.*-SP (example presented in Table 1). Thus, the FI increasing effect of SP is quasi diluted by co-extracted components like neutral glycans and proteins not affecting the FI of Polymer-H. By defining a certain minimum FI increase, the Polymer-H assay could therefore be used as rapid in-process control for the decision, which partial batches can be combined.

**Table 1:** Example: *D.s.*-SP batch obtained by multiple extractions. Increased contents of floridean starch result in lower fluorescence intensity (FI) increases of Polymer-H.

Extract	Floridean starch (%)	Polymer-H FI increase (%)
1st	8.2	105.9 ± 8.3
2nd	9.8	98.1 ± 4.8
3rd	13.1	85.8 ± 2.1
4th	15.9	84.4 ± 0.4

#### 2.2.4. Evaluation of Appropriate FI Increase Limits for the Quality Control of *D.s.*-SP Batches

The investigation of large *D.s.*-SP series revealed that the FI increase of Polymer-H by these SP varies in dependence on their quality and especially correlates with their sulfate content. Lower FI values were usually observed with *D.s.*-SP batches, which had not been isolated by the final protocol or from already multiply extracted algae, further with batches extracted from *Delesseria sanguinea*, which was strongly contaminated with *Mytilus edulis* or harvested in autumn instead of spring and thus containing higher amounts of starch. Additionally, the Polymer-H assay proved to detect instability of *D.s.*-SP.

**Figure 5:**
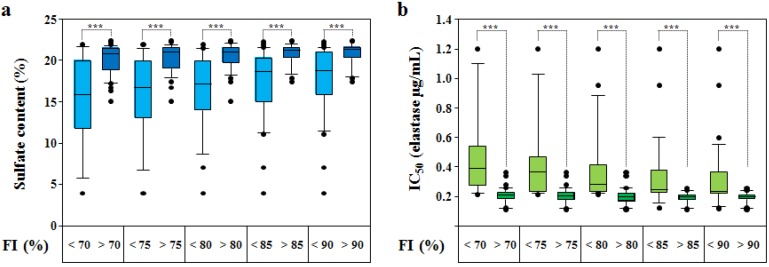
(**a**) Sulfate contents of 57 *D.s.*-SP batches and (**b**) IC_50_ (µg/mL, elastase activity assay) of 55 *D.s.*-SP batches sorted by their fluorescence intensity (FI) increase (%, related to 3.75 µg/mL fondaparinux) at 7.5 µg/mL being either ≤ or > than a certain value (*i.e.*, 70%, 75%, 80%, 85% and 90% of the fondaparinux FI increase at 3.75 µg/mL). Boxplots: median (*black line*), 25th and 75th percentile (end of the boxes), 10th and 90th percentile (*error bars*), outliers (*black points*); *p*-values calculated by Mann-Whitney Rank Sum Test (SigmaPlot 11.0); *** *p* ≤ 0.001.

To utilize the Polymer-H assay as screening method to control the quality of *D.s.*-SP, an appropriate range of FI increase induced by 7.5 µg/mL *D.s.*-SP has to be defined. The upper limit of 105% related to 3.75 µg/mL fondaparinux was appointed by means of the FI increase found with highest-quality *D.s.*-SP batches. Higher FI increase values were only measured with other SP (see Section 2.3.). To assess the lower limit, we tested several FI increase percentages (*i.e.*, 70%, 75%, 80%, 90%, 95%) by dividing the *D.s.*-SP batches into two groups each, *i.e.*, one with lower and the other with higher FI increases and comparing these values with their respective sulfate contents (Figure 5a and Table 2) and IC_50_ in the elastase activity assay (Figure 5b and Table 3).

With a lower FI increase limit of 70%, 42 *D.s.*-SP batches (74%) induced a FI increase >70% whereas with a limit of 90% only 20 batches (35%) exceeded the limit (Figure 5a and Table 2). However, both the median sulfate content and the median IC_50_ of those *D.s.*-SP batches inducing higher FI increases each, only slightly differed (70% *vs.* 90%: 20.6% *vs.* 21.2% and 0.209 µg/mL *vs.* 0.198 µg/mL, Figure 5a,b and Table 2 and Table 3). Greater was the difference in the corresponding coefficients of variation amounting to 8.7% (sulfate content) and 25.4% (IC_50_) for the 70% limit *vs.* 6.1% and 18.2% for the 90% limit. Independent of the FI increase limit, the *D.s.*-SP batches with lower FI increases were very heterogeneous including always samples with sulfate contents and IC_50_ similar to those batches with higher FI increase. To avoid the exclusion of too much *D.s.*-SP batches being adequate concerning other parameters, the limit should therefore not be too high.

Considering the mean FI increase value of 92% ± 7% for *D.s.*-SP_ref_ and the upper FI increase limit of 105%, a lower FI increase limit of 80% seems rational. The 10th/90th percentile sulfate contents and IC_50_ of the batches with FI increases >80% were 18.2%/21.8% and 0.122 µg/mL/0.258 µg/mL, the means ± standard deviations were 20.4% ± 1.6% and 0.199 ± 0.056 µg/mL. The respective coefficients of variation, thus, amounted to 7.8% and 28.1%.

Consequently, a positive result in the Polymer-H assay is a sure criterion for a good quality of the tested *D.s.*-SP batch. But a negative result should not be a final exclusion criterion considering the fact that about one third of the samples with FI increase ≤80% had sulfate contents and IC_50_ within the 10th/90th percentile range of the >80% samples. In such cases additional parameters should be checked, which is anyway necessary in the practice of quality control. Considering the requirements of the European Medicines Agency on the pharmaceutical quality of medicinal products of natural origin (plant extracts), the usually allowed deviation from the mean value (e.g., content of active compounds) is 10%.

**Table 2:** Sulfate contents of 57 *D.s.*-SP batches sorted by their fluorescence intensity (FI) increase (%, see Figure 5a).

FI increase (%)	≤70	>70	≤75	>75	≤80	>80	≤85	>85	≤90	>90
*n*	15	42	18	39	23	34	31	26	35	22
Mean ± SD	15.3 ± 5.3	20.1 ± 1.8	15.7 ± 4.9	20.2 ± 1.7	16.4 ± 4.6	20.4 ± 1.6	17.3 ± 4.3	20.7 ± 1.3	17.6 ± 4.2	20.7 ± 1.3
Median	15.8	20.6	16.8	20.9	17.2	21.0	18.6	21.1	18.8	21.2
10th percentile	5.8	17.3	6.8	17.9	8.7	18.2	11.2	18.3	11.5	18.1
90th percentile	21.6	21.7	21.5	21.7	21.5	21.8	21.6	21.7	21.6	21.9

**Table 3:** IC_50_ (µg/mL, elastase activity assay) of 55 *D.s.*-SP batches sorted by their fluorescence intensity (FI) increase (%, see Figure 5b).

FI increase (%)	≤70	>70	≤75	>75	≤80	>80	≤85	>85	≤90	>90
*n*	13	42	16	39	21	34	29	26	33	22
Mean ± SD	0.476 ± 0.292	0.207 ± 0.053	0.430 ± 0.279	0.205 ± 0.055	0.385 ± 0.255	0.199 ± 0.065	0.342 ± 0.232	0.190 ± 0.039	0.321 ± 0.224	0.193 ± 0.036
Median	0.391	0.209	0.366	0.204	0.281	0.198	0.246	0.198	0.237	0.198
10th percentile	0.222	0.126	0.221	0.122	0.228	0.122	0.162	0.124	0.133	0.122
90th percentile	1.102	0.259	1.026	0.261	0.882	0.258	0.600	0.241	0.552	0.243

#### 2.2.5. Verification of the Defined FI Increase Limits by 8 New *D.s.*-SP Batches

**Figure 6:**
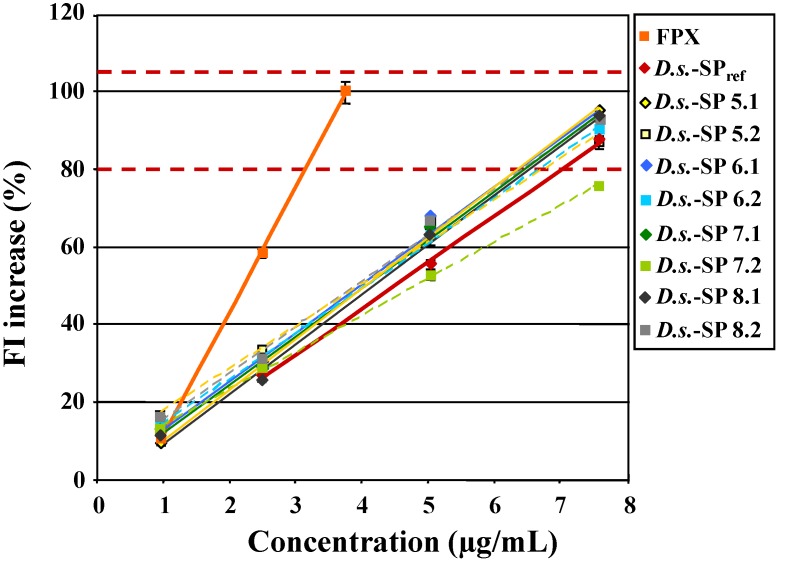
Fluorescence intensity (FI) increase of Polymer-H (7.5 µg/mL in 0.9% NaCl) by 8 *D.s.*-SP batches, fondaparinux (FPX) and *D.s.*-SP_ref_ (x.1/x.2 = 1st/2nd extract of *Delesseria sanguinea* (*D.s.*)).

As proof of concept 8 new *D.s.*-SP batches (see Section 3.3.) were investigated in the Polymer-H assay with the chosen FI increase limits of 80% and 105%. Hereby four concentrations instead of only 7.5 µg/mL (see Section 2.2.2.) were tested, to verify that testing at 7.5 µg/mL is sufficient. As shown in Figure 6, there were only small differences between their FI increase curves and also compared with that of *D.s.*-SP_ref_. With the exception of the second extract *D.s.*-SP 7.2 (75.6% ± 0.3%), all batches met the criterion >80% FI increase at 7.5 µg/mL, which was in line with their high sulfate content (on average > 19.4% ± 0.5%) and good elastase inhibitory activity (on average 0.215 ± 0.006 µg/mL).

The second extracts (dotted lines) induced slightly lower FI increases than the first extracts (solid lines). This matched their some higher starch and protein contents. In contrast, the insufficient FI increase by the second extract *D.s.*-SP 7.2 was confirmed by its low sulfate content (15.8%), its poor elastase inhibitory activity (IC_50_ = 0.276 ± 0.017 µg/mL) and its extremely high content of starch (22.4% *vs.* 6.5% in *D.s.*-SP 7.1). Consequently, the chosen FI limits of 80% and 105% (related to the FI increase by 3.75 µg/mL fondaparinux) measured at 7.5 µg/mL *D.s.*-SP proved to be suitable to control the quality of *D.s.*-SP.

### 2.3. Polymer-H Assay for Identification of D.s.-SP and Distinction from Other Polysaccharides

The observation that *D.s.*-SP, fondaparinux and heparin differed in the shape of their FI increase curves led to the question whether the Polymer-H assay could also be used as a kind of identification assay for *D.s.*-SP and to distinguish them from other SP. For this, six commercially available algae polysaccharides as well as SP batches extracted from three other red algae growing at the “Artificial Reef Nienhagen” were compared with *D.s.*-SP in the Polymer-H assay.

#### 2.3.1. Investigation of Other Algae Polysaccharides in the Polymer-H Assay

The chosen other algae polysaccharides were three prominent examples from red algae, *i.e.*, agarose, κ-carrageenan and λ-carrageenan and three ones from brown algae, *i.e.*, phycarin (a particular laminarin), alginate sodium and fucoidan.

**Figure 7:**
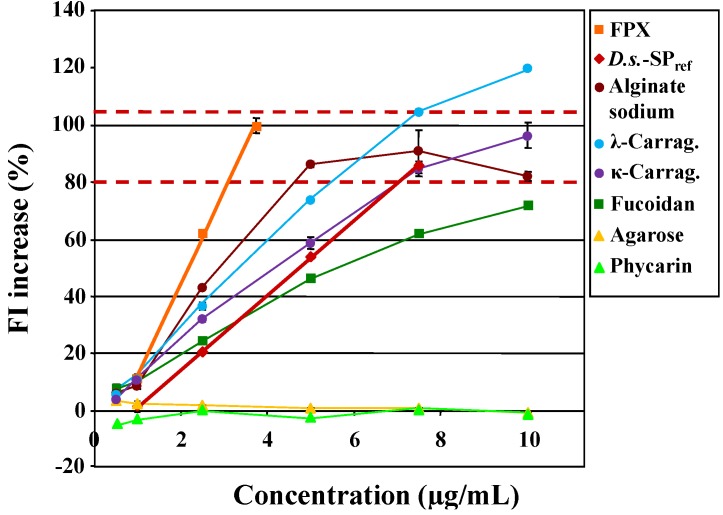
Fluorescence intensity (FI) increase of Polymer-H (7.5 µg/mL in 0.9% NaCl) by six commercially available algae polysaccharides (Carrag. = Carrageenan), fondaparinux (FPX) and *D.s.*-SP_ref_.

The unsulfated phycarin, a β-1,3-glucan, as well as the very low-sulfated agarose, a 3,6-anhydro-α-l-galactose-rich galactan, did not enhance the FI of Polymer-H (Figure 7).

In contrast, the sulfated algae polysaccharides including the unsulfated alginate sodium induced a FI increase. The criterion established for the quality control of *D.s.*-SP batches (see Section 2.2) failed to allow a clear distinction, since apart from that of fucoidan the FI increases at 7.5 µg/mL were within the 80%–105% range of that induced by 3.75 µg/mL fondaparinux. Therefore, concentration-dependent curves had to be considered (Figure 7, Table 4). Within the meaning of the concept of a screening assay, the concentration range was restricted to the linear range of *D.s.*-SP_ref_ (1–7.5 µg/mL).

The slope of the linear parts of the FI curves of both the three sulfated algae polysaccharides and *D.s.*-SP_ref_, heparin and fondaparinux degree of sulfation dependently increased (Figure 7, Figure 2 (heparin), Table 4), what confirms the degree of sulfation dependence found with semi-synthetic glucan sulfates [25].

The only exception to this was the relatively steep slope of the unsulfated alginate sodium, whereby the FI increased only up to 5 µg/mL. At higher concentrations, FI increase retained roughly constant and was lower than the usually found maxima of sulfated glycans. An explanation for the interaction with Polymer-H might be that alginate sodium exclusively consists of negatively charged uronic acid and therefore has a degree of charges of 1.0. The lower maximum FI increase could be interpreted as an indication that sulfate groups have a stronger tendency to bind to Polymer-H than carboxyl groups.

**Table 4:** Polymer-H assay-based test concept for identification and differentiation of sulfated poly- and oligosaccharides. By testing four concentrations, the Polymer-H assays allows distinction between structurally different sulfated poly- and oligosaccharides by means of the three parameters FI increase at 7.5 µg/mL, linear range and slope of the line. By inclusion the data of a reference sulfated polysaccharides (SP), the considered SP can be identified. For identification of closely related SP, additional characteristics such as the sulfate content and the elastase inhibitory activity (IC_50_) have to be included.

Substances	Polymer-H assay						Sulfate content	Elastase inhibition (IC_50_)	Distinction from *D.s.*-SP by additional parameters
	Mean FI increase at 7.5 µg/mL (%)	Linearity ^1^ (1.0 to 7.5 µg/mL)	slope of the line ^2^ (rel. to *D.s.*-SP_ref_)	Distinction from *D.s.*-SP					
	1	2	3	1	2	3	(% m/m)	(µg/mL)	PolyH/other
***D.s.*-SP**	80–105	yes	1.00 ± 0.11	-			20.4 ± 1.6	0.20 ± 0.02	-
***D.s.*-SP_ref_**	92 ± 7	yes	1.00	-			20.5	0.19	-
*Structurally different oligo*-*and polysaccharides*									
**Fondaparinux**	110	no	2.47	+	+	+	41.5	>10	+
**Heparin**	107	no	2.06	+	+	+	27.7	0.31	+
**Phycarin**	0	no	0.0	+	+	+	0.0	>25	+
**Agarose**	0	no	0.0	+	+	+	≤0.2	>6.25	+
**Fucoidan**	63	no	0.68	+	+	+	20.8	0.69	+
**Alginate sodium**	91	no	1.47	−	+	+	0.0	>2.5	+
**λ-carrageenan**	106	(yes)	1.08	(+)	−	− ^3^	44.0	0.22	+ ^3^/SC
**κ-carrageenan**	85	(yes)	0.85	−	−	(+) ^3^	20.7	>6.25	+ ^3^/IC_50_
*Red algae polysaccharides structurally related to D.s.*-*SP*									
***D.s.*-SP 7.1**	93	yes	0.92	−	−	−	20.2	0.19	-
***P.r.*-SP**	113	yes	1.18	+	−	(+) ^3^	21.0	0.19	+
***P.p.*-SP**	99	yes	1.01	−	−	−	16.7	0.45	−/SC, IC_50_
***C.t.*-SP**	72	yes	0.73	+	−	+	16.0	0.18	+

^1^ linearity: *r* > 0.997; ^2^ slope of the line in the range of 1.0–7.5 µg/mL, except for fondaparinux (FPX) and heparin: 1.0–3.75 µg/mL, fucoidan and alginate sodium: 1.0–5.0 µg/mL; ^3^ by the FI values at 2.5 and 5.0 µg/mL, distinction from *D.s.*-SP is possible.

In addition to the FI increase at 7.5 µg/mL, a second criterion to distinguish different polysaccharides from each other is the slope (Table 4). In this respect, *D.s.*-SP significantly differed from fondaparinux, heparin, fucoidan and alginate sodium as well as of course from phycarin and agarose, but less clearly from κ-carrageenan and λ-carrageenan with slopes of 0.85 and 1.08 in relation to 1.00 ± 0.11 of *D.s.*-SP (since only one batch of κ- and λ-carrageenan, respectively, was tested).

A third criterion is the respective linear range of the FI curves. In contrast to *D.s.*-SP, the FI curves of fondaparinux, heparin, fucoidan and alginate sodium were not linear in the considered range from 1.0 to 7.5 µg/mL, whereas those of the two carrageenans were almost linear (Table 4).

Nevertheless, the two latter can be differentiated from *D.s.*-SP by a fourth criterion, namely the extent of the FI values at the various concentrations. In fact, their curves appeared to be roughly parallel shifted to lower concentrations. Compared to *D.s.*-SP_ref_ (sulfate content of 20.5%), the FI values for λ-carrageenan (sulfate content of 44.0%) were 82%, 39%, and 23% higher at 2.5, 5.0, and 7.5 µg/mL and those for κ-carrageenan (sulfate content of 20.7%) 53% and 9% higher at 2.5, and 5.0 µg/mL. The stronger effects of κ-carrageenan despite its similar sulfate content indicate that the FI increasing potency is additionally influenced by other structural parameters, which still need to be elucidated.

Taken together, with the three criteria FI increase at 7.5 µg/mL, slope of the FI line and linearity range, *D.s.*-SP could be clearly distinguished from the other glycans. For this, testing of four concentrations of the SP in the linear range of *D.s.*-SP or the reference for another SP of interest, respectively, turned out to be sufficient. Consequently, the Polymer-H assay has an excellent negative predictability.

The result that the three red algae-derived, high-molecular mass sulfated galactans *D.s.*-SP_ref_, λ- and κ-carrageenan could only be differentiated by a fourth criterion, *i.e.*, FI increases at other concentrations, provokes the question whether structurally even more related SP are still distinguishable from each other.

#### 2.3.2. Investigation of Sulfated Xylogalactans from Various Red Algae in the Polymer-H Assay

**Figure 8:**
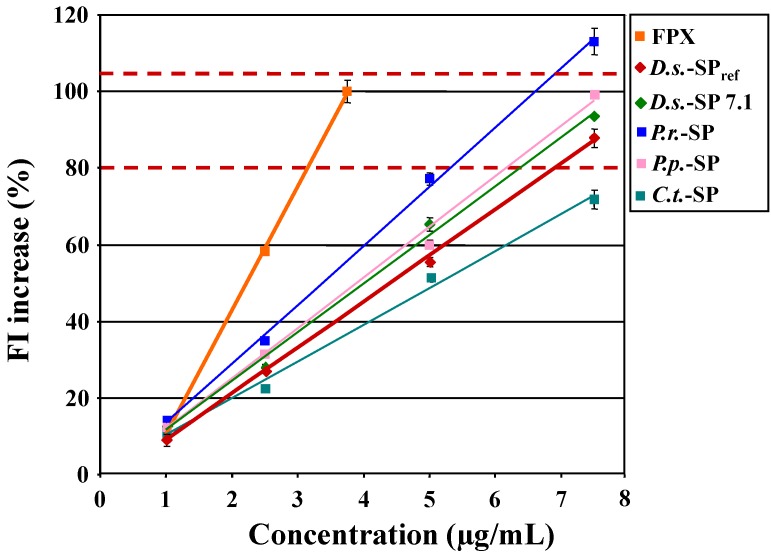
Fluorescence intensity (FI) increase of Polymer-H (7.5 µg/mL in 0.9% NaCl) by SP from four red algae collected at the “Artificial Reef Nienhagen”, fondaparinux (FPX) and *D.s.*-SP_ref_.

To clarify whether the Polymer-H assay allows differentiating between closely related SP, sulfated xylogalactans from three other red algae were compared with *D.s.*-SP (Figure 8). The selected algae were *Phycodrys rubens* (*P.r.*), *Phyllophora pseudoceranoides* (*P.p.*) and *Coccotylus truncatus* (*C.t.*), which all grow at the “Artificial Reef Nienhagen”.

To exclude any environmental and seasonal influences on the composition of the SP, batches of the three algae as well as *Delesseria sanguinea* were harvested at the reef at the same day in May 2011. The algae material was extracted according to the protocol for *D.s.*-SP. The four SP batches (7.5 µg/mL) increased the FI of Polymer-H in the following order: *C.t.*-SP (72%) < *D.s.*-SP_ref_ (88%) < *D.s*.-SP 7.1 (93%), *P.p.*-SP (99%) < *P.r.*-SP (113%) (Figure 8, Table 4). Consequently, *C.t.*-SP and *P.r.*-SP could already be distinguished from *D.s.*-SP by this basic Polymer-H assay parameter, but not *P.p.*-SP. Comparing *D.s*.-SP 7.1 with *D.s.*-SP_ref_, the new batch met the requirement according to Section 2.2.

In contrast to the other tested SP, these four structurally related xylogalactans had the same linear range, but differed in the slope of the FI curves. The slopes did not correlate with the sulfate contents: despite similar sulfate contents that of *P.r*.-SP was larger than that of *D.s.*-SP 7.1 and that of *P.p*.-SP larger than that of *C.t.*-SP. This might be due to differences in the detailed structures of these xylogalactans including e.g., distinct substitution with pyruvyl groups, which increase the negative charge density and contribute to interactions with Polymer-H (see alginate sodium; manuscript in preparation).

To sum up, related SP can partly be differentiated from each other by the Polymer-H assay, but as shown by *D.s.*-SP and *P.p.*-SP this method is not always sufficient. In addition, the fact that SP from algae usually display certain natural variability [19] may hamper a clear differentiation between structurally related SP.

#### 2.3.3. Test Concept for Identification of *D.s.*-SP and Distinction from Other Sulfated Glycans

The Polymer-H assay proved to have an excellent negative predictability, but only a moderate positive predictability. As demonstrated by the four red algae-derived sulfated xylogalactans, discrimination between structurally related sulfated glycans can be tricky and may only be possible using high-sophisticated analysis (NMR and GC-MS).

For some purposes like an initial screening, it is, however, of interest to have simpler and cheaper methods to differentiate also between similar SP. Therefore, we evaluated a concept similar to that of the pharmacopoeias, where the identification of a compound generally requires more than one test. For this, it was checked whether a combination of Polymer-H assay with two established routine tests, namely sulfate determination and elastase activity assay, overcome the moderate positive predictability of the fluorescence method. Indeed, *P.p.*-SP and *D.s.*-SP, which proved to be indiscernible by the Polymer-H assay, significantly differed in their elastase inhibitory activity (IC_50_ = 0.45 *vs.* 0.20 ± 0.02 µg/mL) and their sulfate content (16.7% *vs.* 20.4% ± 1.6%, Table 4). Contrary to this, *P.r.*-SP and *D.s.*-SP had similar sulfate contents and elastase inhibitory activities and could only be distinguished by their effects on Polymer-H (Table 4). In the case of the two exemplarily investigated carrageenans, a differentiation from each other was possible by the Polymer-H assay, but that from *D.s.*-SP demanded confirmation. For λ-carrageenan, this was given by the sulfate content (44.0% *vs.* 20.4% ± 1.6%), and for κ-carrageenan by the elastase inhibitory activity (>6.25 µg/mL *vs.* 0.20 ± 0.02 µg/mL).

Moreover, an advantage of the Polymer-H assay is that it detects not only sulfated glycans, but also responses to anionic polysaccharides. In this way, it enables for example to distinguish between agarose and the cheaper alginate sodium [12] without application of typical identification assays for these two phycocolloids. As demonstrated by these examples, the combination of concentration-dependent response in the Polymer-H assay (four concentrations), sulfate content and elastase inhibitory activity represents an efficient and simple concept for the differentiation of various sulfated glycans. By inclusion of corresponding reference compounds like *D.s.*-SP_ref_ in this study, the investigated sulfated glycans can be identified. Whereas the sulfate content is generally a useful test parameter for sulfated glycans, the elastase inhibitory effect can of course be replaced by any other characteristic method relevant for the application of the examined compounds (e.g., viscosity, gel formation, anticoagulant activity).

Depending on the purpose of the testing, this concept may be appropriate or has to be supplemented by orthogonal methods delivering more information on the structural composition of the sulfated glycans. For example, the identification of the drug substance heparin sodium according to the monograph of the European Pharmacopoeia requires not only the determination of anticoagulant activity, but also ^1^H-NMR analysis, strong ion exchange liquid chromatography and atomic absorption spectrometry [30]. In contrast, for the identification of alginic acid two simple gelling assays and an unspecific color reaction with dihydroxynaphthalene are thought to be sufficient [31].

## 3. Experimental Section

### 3.1. Polymer-H

The sensor Polymer-H for sulfated glycans has been synthesized as previously described [26]. In short, first the three comonomer units, *i.e.*, methacrylamide derivatives of (1) the dansyl; (2) the *ortho*-aminomethylphenylboronic acid and (3) the ethylammonium moiety were prepared and then subjected to conventional radical copolymerization resulting in Polymer-H.

### 3.2. Heparin, Fondaparinux and PS3

Unfractionated heparin (heparin, from porcine intestinal mucosa) and fondaparinux sodium (Arixtra^®^) were kind donations from Novartis (Nürnberg, Germany) and Glaxo Smith Kline (Notre Dame de Bondville, France), respectively. The characteristic value for mass-average *M*_r_ of heparin is 15,000 and the *M*_r_ of fondaparinux is 1728. The degrees of sulfation of heparin and fondaparinux are 1.20 and 1.60, respectively.

PS3, a structurally defined linear β-1,3 glucan sulfate with *in vivo* anti-inflammatory activity (US patent No. US7008931-B2, produced under GMP conditions, purity >99%) was synthesized as previously described [32,33]. It has a *M*_r_ of 10,000 and a degree of sulfation of 2.2.

### 3.3. Sulfated Polysaccharides Fraction of Delesseria sanguinea (D.s.-SP)

*D.s.*-SP were isolated from the red alga *Delesseria sanguinea* (Hudson) Lamouroux using a step-wise optimized procedure [18]. The algae material was collected from the large “Artificial Reef Nienhagen” in the southwestern Baltic Sea (geographical position: φ = 54°10.50′N; λ = 11°56.60′E), from 2006 to 2011. *D.s.*-SP consist of a homogeneous fraction of branched sulfated xylogalactans with an average *M*_r_ of 142,000 (gel filtration chromatography with detection by multi-angle laser light scattering). The average degree of sulfation of *D.s.*-SP isolated with the finally optimized procedure amounts to 0.75 ± 0.11, whereby the degree of sulfation of the pure xylogalactan is meant.

*D.s.*-SP_ref_ was chosen as reference compound. It was isolated from *Delesseria sanguinea* material, which was collected from the “Reef Nienhagen” in April 2008, according to the finally optimized procedure. Characteristics: 20.5% sulfate content, degree of sulfation of xylogalactan 0.74, 10.5% protein content, 4.9% floridean starch and IC_50_ (elastase) 0.186 ± 0.004 µg/mL.

Overall, a heterogeneous pool of 65 *D.s.*-SP batches was included in this study, whereby 6 batches were extracted from *Delesseria sanguinea* harvested at other locations than the reef (see Section 2.2.1), 57 batches (including the latter) served for the investigation of the dependence of the FI increase on the sulfate and protein content (Section 2.2.2) and the eight newest batches were used for the verification of the defined FI increase limits (Section 2.2.5).

These 65 *D.s.*-SP batches were extracted from 12 different *Delesseria sanguinea* batches, which were harvested in spring, summer and autumn of the years 2006, 2009, 2010, and 2011, respectively.

Most of the investigated *D.s.*-SP batches were extracted from purified (“+”) *Delesseria sanguinea*, which means the algae material was manually purified from epiphytic and epizoic contaminants, in fact, primarily *Mytilus edulis* L. *D.s.*-SP batches from unpurified *Delesseria sanguinea* are (“−”)-labeled.

The sulfate content of the 65 *D.s.*-SP batches (including the desulfated batches) investigated in this study ranged from 3.9% to 22.4%. The protein contents of the 65 batches ranged from 4.8% to 35.6% and their IC_50_ in the elastase activity assay from 0.113 to 1.201 µg/mL (2 desulfated batches: IC_50_ > 25 µg/mL). For the experiments described in Section 2.2.3., 2 *D.s.*-SP batches were desulfated by incubation of 5 mg/mL *D.s.*-SP solved in a mixture of 1 mL/10 mg pyridine and dimethylformamide (ratio double distilled water:dimethylformamide = 1:9) for 24 h at 90 °C followed by dialysis and lyophilization. Moreover, four batches were obtained by multiple extractions, this means the same algae material was extracted not only once (1st extraction), but four times using always fresh extraction solvent (2nd, 3rd, 4th extraction).

The 8 *D.s.*-SP batches for the verification of the defined FI increase limits (see Section 2.2.5) were extracted from *Delesseria sanguinea*, harvested in May 2011. The algae material was divided into four parts, which were differently processed and stored before a two times extraction (1st and 2nd extraction) each. The extracted *Delesseria sanguinea* were freshly harvested and stored in sea water (*D.s.*-SP 5.1 and 5.2), lyophilized (*D.s.*-SP 6.1 and 6.2), dried at 50 °C in a cabinet drying (*D.s.*-SP 7.1 and 7.2) or stored in EtOH (99% *v*/*v*) for seven weeks (*D.s.*-SP 8.1 and 8.2), respectively.

### 3.4. Other Algae Polysaccharides

Three SP isolated from three other red algae growing at the “Artificial Reef Nienhagen” were isolated according to the procedure used for *D.s.*-SP: (1) *C.t*.-SP from *Coccotylus truncatus* (*C.t.*); (2) *P.p.*-SP from *Phyllophora pseudoceranoides* (*P.p.*) and (3) *P.r*.-SP from *Phycodrys rubens* (*P.r.*).

Fucoidan (from *Fucus vesiculosus*, F5631), agarose (A9539), alginate sodium (A0682) as well as λ-carrageenan (22049) and κ-carrageenan (22048) were purchased from Sigma (St. Louis, MI, USA). The β-1,3-glucan phycarin (from *Laminaria digitata*) was obtained from Goemar Laboratories (St. Malo, France).

### 3.5. Polymer-H Fluorescence Assay

The following method was used for the tests described in Section 2.2 and Section 2.3.

The sample compounds were dissolved in 0.9% aqueous NaCl. Sample aliquots of 180 µL were mixed with 20 µL Polymer-H solution (75 µg/mL) in a 96-well-microplate (MaxiSorp^®^, nunc GmbH & Co.KG, Schwerte, Germany). After equilibration for 10 min at room temperature, the fluorescence signals were read out by top reading (λ_ex_ = 320 ± 10 nm, λ_em_ = 510 ± 10 nm) using the microplate reader FLUOstar Omega (BMG LABTECH GmbH, Ortenberg, Germany). Fondaparinux and *D.s.*-SP_ref_ dissolved in 0.9% aqueous NaCl were used for calibration and as reference compound, respectively. As controls, the basic FI of Polymer-H (in 0.9% aqueous NaCl) and the FI increase of Polymer-H by 3.75 µg/mL fondaparinux were measured and represent the 0 and 100% values. The FI values of the samples obtained by subtracting the basic FI (Polymer-H dissolved in 0.9% NaCl) from the measured FI values. In some cases, these resulting values are indicated as FI increase (%) in relation to the FI increase by fondaparinux (100%).

### 3.6. Analytical Methods

The protein and sulfate contents (%) of the SP were calculated by using the nitrogen and sulfur contents, respectively, determined by elementary analysis of the dried SP. The elementary analysis of carbon, hydrogen, nitrogen and sulfur was performed by the HEKAtech CHNS Analyser (HEKAtech GmbH, Wegberg, Germany, calibrator: sulfanil amide). After gas chromatographic separation (carrier gas: Helium), the respective analyte gases were detected in a thermal conductivity detector. Based on the sulfate, protein and glucose content, the degree of sulfation of the SP was calculated.

The quantitative monosaccharide composition of the SP was determined by gas-liquid chromatography of alditol acetates as previously described [18]. In the case of *D.s.*-SP, the glucose content was used to calculate the content of floridean starch.

### 3.7. Elastase Activity Assay

For the determination of the elastase inhibitory activity, the following fluorescence microplate assay was used [34]: An aliquot of 25 µL of the sample compound (dissolved in 0.9% aqueous NaCl) was mixed with 25 µL of Tris buffer (50 mM Tris, 155 mM NaCl, pH 8.3) and 25 µL of human PMN elastase (Calbiochem, Merck KGaA, Darmstadt, Germany, *c* = 100 nM, diluted in sodium acetate buffer: 50 mM sodium acetate, 200 mM NaCl, 1% BSA, pH 5.5). After 5 min incubation at 37 °C, 25 µL of substrate solution (I-1270, Bachem, Germany, *c* = 3 mM, diluted in Tris buffer) were added. After incubation for another 5 min at 37 °C, the fluorescence was measured (λ_ex_ = 370 ± 10 nm, λ_em_ = 450 ± 10 nm). As 100% control, NaCl 0.9% solution instead of inhibitor solution was used. The fluorescence signal of NaCl 0.9% instead of inhibitor and pure sodium acetate buffer instead of elastase solution served as blank (0 control). The fluorescence values obtained by subtracting the blank from the measured values were used to calculate the elastase inhibition (%) in relation to the 100% control. To determine the inhibitor concentrations for 50% inhibition (IC_50_), the data of the linear range of the concentration-dependent curves were analyzed with Sigma Plot 11.0 (Systat Software GmbH, Erkrath, Germany).

### 3.8. Statistical Analysis

All measurements were done in duplicate and repeated on different days. The data are presented as means ± standard deviations. To evaluate the dependence of the FI increase in the Polymer-H assay on the sulfate and protein content of the SP as well as to establish appropriate FI increase limits by means of the sulfate content, the data of 57 *D.s.*-SP batches (including the two desulfated batches) were analyzed. To establish appropriate FI increase limits by means of the IC_50_ values, the analysis included the data of 55 of these batches (without the two desulfated batches). The statistical analysis was performed by the Mann-Whitney rank sum test (program Sigma Plot 11.0), *p* ≤ 0.001 was considered statistically significant. Outliers are illustrated as black points.

## 4. Conclusions

The results of this study demonstrate that the Polymer-H assay represents a simple and rapid method not only for quantification of SP [25], but also for characterization of SP, prone to variability, including their identification and differentiation (in combination with other assays). This fluorescence microplate assay is based on the fact that sulfated glycans concentration-dependently increase the FI of the sensor Polymer-H, whereby it has a higher sensitivity and better reproducibility than colorimetric assays like the dimethylmethylene blue test [35].

As exemplarily revealed by large series of *D.s.*-SP batches from the red algae *Delesseria sanguinea*, the assay is well suitable to screen the quality of SP from algae and to control the batch-to-batch consistency. It showed to detect *D.s.*-SP variations due to different isolation procedures as well as due to the extraction of *D.s.* thalli strongly contaminated with epibionts, harvested at different locations or at different seasons, respectively. Further, it proved suitable as stability test for SP. For such quality control purposes, testing of just one concentration (e.g., 7.5 µg/mL *D.s.*-SP) of the sample together with corresponding reference compounds turned out to be sufficient. 

By comparing the FI data of 57 (overall 65) *D.s.*-SP batches with their sulfate contents and elastase inhibitory activities as two typical quality parameters, upper and lower limits for the FI values of high-quality *D.s.*-SP were established. Analogously, adequate FI ranges can be elaborated for any other SP, whereby the chosen characteristics of the SP depend on its intended use.

In addition to the use for quantification and quality assessment of SP, the Polymer-H assay turned out to allow distinguishing between different sulfated glycans. Although sulfated glycans generally increase the FI of Polymer-H, their concentration-dependent FI increase profiles showed to differ. This observation suggests that the FI increase of Polymer-H by sulfated glycans is additionally influenced by other structural parameters than their degree of sulfation. By using corresponding reference compounds, the Polymer-H assay may therefore be applied as identification assay with high negative predictability. In the case of closely related compounds, the combination with other assays is however necessary.

In conclusion, the Polymer-H assay is supposed to represent a rapid, simple and cheap test not only for quantification, but also for an initial quality screening of SP. An analytical method for SP with such performance characteristics seems desirable, since especially algae polysaccharides are known to considerably vary in their chemical composition and purity due to numerous environmental factors as well as isolation process-related parameters [19]. Depending on the issue and the intended use of the SP (e.g., as active agent or excipient in medicinal products), the Polymer-H assay does certainly not replace comprehensive quality assessment. However, in situations and locations, where the required high-sophisticated methods [22] are not *ad hoc* available, too elaborate, or too expensive, it may represent a helpful tool.
